# Antimicrobial Activity of a Neem Cake Extract in a Broth Model Meat System

**DOI:** 10.3390/ijerph10083282

**Published:** 2013-08-02

**Authors:** Paola Del Serrone, Marcello Nicoletti

**Affiliations:** 1 Agricultural Research Council, Research Centre Animal Production and Genetic Improvement, Via Salaria 31 Km 26, Monterotondo 00015 RM, Italy; 2 University of Rome Sapienza, Pia.le Aldo Moro 5, Rome 00185, Italy; E-Mail: marcello.nicoletti@uniroma.it

**Keywords:** antimicrobial activity, *Azadirachta indica*, neem cake, zoonotic and spoilage bacteria, meat quality, safety control

## Abstract

This work reports on the antimicrobial activity of an ethyl acetate extract of neem (*Azadirachta indica*) cake (NCE) against bacteria affecting the quality of retail fresh meat in a broth model meat system. NCE (100 µg) was also tested by the agar disc diffusion method. It inhibited the growth of all tested microorganisms. The NCE growth inhibition zone (IZ) ranged 11.33–22.67 mm while the ciprofloxacin (10 µg) IZ ranged from 23.41–32.67 mm. There was no significant difference (*p* ≤ 0.05) between the antimicrobial activity of NCE and ciprofloxacin *vs. C. jejuni* and *Leuconostoc* spp*.* The NCE antibacterial activity was moreover determined at lower concentrations (1:10–1:100,000) in micro-assays. The percent growth reduction ranged from 61 ± 2.08–92 ± 3.21. The higher bacterial growth reduction was obtained at 10 µg concentration of NCE. Species-specific PCR and multiplex PCR with the DNA dye propidium monoazide were used to directly detect viable bacterial cells from experimentally contaminated meat samples. The numbers of bacterial cells never significantly (*p* ≤ 0.05) exceeded the inocula concentration used to experimentally contaminate the NCE treated meat. This report represents a screening methodology to evaluate the antimicrobial capability of a herbal extract to preserve meat.

## 1. Introduction

The use of herbal extracts in food preservation is not a novelty and it is increasing, however more herbal extracts need to be tested and validated with alternative methods. Microbial contamination of highly perishable products such as fresh meat is caused mostly by improper livestock rearing and processing practices, unclean processing tools, environments and defective worker hygiene. Furthermore, microbial species which may survive or proliferate on meat after slaughtering and sectioning can be haematogenically spread (diapedesis) from the gastrointestinal system to muscles. Causes of the spreading are the shocks caused to the animal during the process of culling and transport to the slaughterhouse.

Meat contamination results in changes in appearance and odor during prolonged storage. Often Lactic Acid Bacteria (LAB) are responsible of discoloration, slime formation, off-odors and off-flavors as the result of their metabolic activity. However, contamination can also be present without such sensory evidence, requiring deep and adequate controls.

The increasing incidence of foodborne diseases, coupled with the resultant social and economic implications, causes a constant impulse to produce safer feed and food and to develop new natural antimicrobial agents [1,2,3]. As a matter of fact, the uncontrolled overuse of antibiotics as common feed supplements could lead to increased numbers of antibiotic-resistant bacteria, and could ultimately compromise treatments of bacterial infections in humans. There is, also, the possibility to transfer the antibiotic-resistant microorganisms into humans both directly via food chain and indirectly by animal waste spread throughout fields.

In 1998 the EU health ministers started to ban antibiotics widely used to promote animal growth. In 2006 EU banning regulations in animal feed became effective. In the USA, the food and animal industries have so far successfully blocked most legislation to ban antibiotics in feed. The reality is that most countries underestimate or avoid the problem. On the other hand, the problem of safe preservation in the food industry has grown to be more complex. Today’s products require longer shelf-life and greater assurance of protection from microbial contamination. International authorities, including the WHO, described the possible scenario of a Post-Antibiotic Era, based on the worrying conjuncture of bacteria becoming resistant to every kind of antibiotic. The inexorable rise of multidrug-resistance needs the exploration of new strategies, including urgent and corrective action. Future fight to uncontrolled bacteria may be based on the shift from the single molecule to the multiple arsenals of natural products revised and validated by technology. The exploration of plant-derived antimicrobials should be an innovative way to find alternative substances to antibiotics today [4,5,6].

Neem (*Azadirachta indica* A. Juss) is a monumental tree of Meliaceae family coming from the Indian subcontinent. Actually, importance and distribution of neem is increasing all over the World due to its beneficial properties, as reported by WHO/UNEP1989 [7]. Neem is considered an effective source of environmentally powerful natural pesticides and considered to be one of the most promising trees of the 21st century for its great potential in pest management, environmental protection and medicine. The International Scientific Community included the neem tree in the top ten list of plants to be studied and used for the sustainable development of the planet and the health of living beings. Various derivatives of neem have showed relevant uses in the toiletries and pharmaceuticals markets, the manufacture of agricultural implements and furniture, cattle and poultry feeds, and nitrification of soils for various agricultural crops [8,9]. The multipurpose character of neem is directly derived from its complex chemical composition, subjected to high variability, also reflected into the composition of derived products. Among the many products obtained from the seeds, neem oil is the most commercially relevant.

Neem cake is the waste by-product remaining after the oil extraction processes. Neem is considered devoid of toxicity, as tested also by the ancient traditional use. Neem cake has been successfully utilized as livestock feed for growing goats [10]. The antimicrobial activity of *A. indica* seed oil was also investigated [11,12]. The purpose of this work was to evaluate the antimicrobial activity of a neem cake ethyl acetate (CH_3_COOCH_2_CH_3_, EtOAc) extract (NCE) against bacterial populations that badly affect quality of retail fresh meat. The antimicrobial activity of NCE was evaluated in three steps: growth inhibition zone on solid medium, percent growth reduction in liquid medium, detection of the surviving microorganisms in experimentally inoculated meat after treatment with NCE simulating an abusive refrigerated storage. In the present study molecular biology techniques were employed in addition to the use of classical methodologies to evaluate the antibacterial activity of NCE. Among the molecular biology techniques, PCR and multiplex PCR with PMA^TM^ dye of DNA, are very useful to specifically detect viable cells of microorganisms affecting meat quality. The use of PMA^TM^ is a valid alternative method to assess bacterial growth or its inhibition instead of the colorimetric methods using a tetrazolium salt. They are reported as good indicators of bacterial growth, but difficulties arise because of the autofluorescence, salt reduction and the antioxidant properties of plant products, especially for XTT (3′-(1-[(phenylamino)-carbonyl]-3,4-tetrazolium)-bis(4-methoxy-6-nitro)- benzenesulfonic acid hydrate), TTC (2,3,5-triphenyltetrazolium chloride), and resazurin (7-hydroxy-3*H*-phenoxazin-3-one-10-oxide) [13,14]. Furthermore, tetrazolium salts are not suitable to assess the growth of microaerophilic bacteria [15].

The real-time polymerase chain reaction (qPCR) to amplify and simultaneously quantify targeted microorganisms’ DNA could also be used, but this last technique requires equipment which cost is tenfold higher than that of a thermal cycler and higher specialist knowledge to set the protocols for its use. Instead PCR and multiplex PCR are cheaper, less time consuming and the results are easily readable in comparison with qPCR and microbiological methods. These characteristics allow one to perform rapid and efficacious massive screening of samples as for safety control surveys.

## 2. Materials and Methods

### 2.1. Plant Material

**Figure 1:**
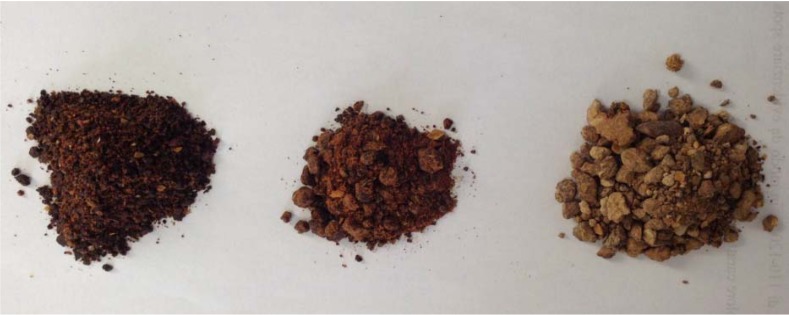
Samples of commercial neem cakes. NCE was obtained from the product in the middle.

A commercial deoiled neem cake produced by Neem Green [16] was used as test starting material (Figure 1). Neem cake was the by-product of the manufacture of neem oil, obtained by cold pressing neem kernels from handpicked and cleaned neem fruits and seeds. NCE was obtained by direct extraction of the dried neem cake with ethyl acetate (1:10 wt/v) at room temperature for 24 h [17]. Thereafter the resulted NCE was dried and kept at −4 °C until further uses.

### 2.2. Test Microorganisms

The microbial strains Campylobacter jejuni, Carnobacterium spp., Lactobacillus curvatus, Lactobacillus sakei and Leuconostoc spp., were used to test the antibacterial activity. Carnobacterium spp., Lactobacillus curvatus, Lactobacillus sakei and Leuconostoc spp. represent the most common spoilage agents of meat while Campylobacter jejuni is one of the most relevant foodborne pathogens.

These bacterial strains were isolated in a previous study [18,19] and then maintained in Microbank^TM^ vials at −70 °C. *Escherichia coli*, American Type Culture Collection (ATCC^®^) 51813^TM^, *Enterococcus faecalis* ATCC^®^ 7088^TM^ and *Staphylococcus aureus* ATCC^®^ 6538^TM^ were also used as control strains. To prepare working cultures, stock cultures were standardized through two successive 24 h growth cycles in the appropriate broth (BHI Difco, Buccinasco, MI, Italy) at 20 °C without agitation.

### 2.3. Chemical Analysis

Total composition of the NCE was checked by High Performance Thin Layer Chromatography (HPTLC). Filtered solutions in MeOH were applied to 60 silica gel glass-backed plates (Merck, Darmstadt, Germany) by a Camag Linomat IV sample applicator (Camag, Basel, Switzerland) with nitrogen flow in 4:6 toluene/ethyl acetate and examined under white light, and 254 nm and 366 nm UV light. Nortriterpene (limonoid) composition was obtained on a High Performance Liquid Chromatography apparatus (Perkin Elmer, Waltham, MA, USA) equipped with a 20 μL injection loop, a Restek C 18 II Pinnacle column (250 × 46 mm, 5 μm particles, Restek Corporation, Bellefonte, PA, USA), stationary phase, flow rate: 1.00 mL·min^−1^, UV-VIS detector: 214 nm, elution program: 8 min isocratic, 45% CH_3_CN/55% water; 22 min linear gradient to 100% CH_3_CN; 10 min isocratic 100% CH_3_CN) and a HPTLC apparatus (Camag, Muttenz, Switzerland) with HPTLC silica gel 60 F_254_ MS-grade (Merck Millipore, Darmstadt, Germany) in chloroform/methanol 9:1 v/v as mobile phase).

### 2.4. Antibacterial Activity in vitro Experiment

NCE was dissolved in Tween^®^ 80 (VWR International PBI Srl, Milano, Italy, 1 mg/mL) under agitation. The solution was sterilized by filtration through a 0.22 μm sterilizing Millipore express filter (Millex-GP, Bedford, OH, USA). Whatmann filter paper (n°1) discs of 6 mm diameter were impregnated with 100 µL of NCE solution. Discs impregnated with 100 µL of Tween and 10 µL (wt/v) of antibiotic (ciprofloxacin hydrochloride monohydrate, 1 mg/mL, Bayer, Milano, Italy) were used as negative and positive control, respectively.

Two discs were prepared for each sample. They were evaporated at 37 °C for 24 h. Bacterial suspension (100 µL), standardized by adjusting the optical density to OD 1 at 600 nm (Shimadzu UV-120-01 spectrophotometer, Shimadzu, Cinisello Balsamo, MI, Italy), was used to inoculate by flooding the surface of suitable agar media for each microorganism considered at their proper growth conditions (see Section 2.5.4). Excess liquid was air-dried under a sterile hood and the impregnated discs were applied at equidistant points on top of the agar medium.

The plates were done in triplicates for each organism and the experiment was performed twice. Plates were checked after incubation at 35 °C and 42 °C for 48 h. Antimicrobial activity was measured as diameter (mm) of the inhibition zone (IZ) around the disc. The results were recorded as means ±SD of the duplicate experiment.

The antibacterial activity of NCE against the bacteria was also evaluated in micro-assays using conventional sterile polystyrene microplates. Each well of the microplate was filled with 100 μL of sterile suitable liquid media for each microorganism considered, 50 μL of inoculums (see Table 4) and amounts of extract at lower concentrations (1:10–1:100,000) were added. Control treatment without NCE was used in the experiment. The microplates were incubated at 37 °C for 24 h. Bacterial growth was determined by OD reading at 630 nm/10 mm pathlength with an ELISA microplate reader (Dynatech ML-3000, Pina de Ebro, España). Bacterial cell concentration was transformed to cells/mL using the reference curve equation.

The reference curve was constructed by diluting 1:100 each bacterial species. Counting the number of bacterial cells of an aliquot of this dilution was done using a Neubauer chamber (Celeromics, Vedano al Lambro, MI, Italy). Finally, cell concentrations were transformed to a percentage of bacterial inhibition. The percentage of bacterial growth reduction (GR%) was estimated using as reference the control treatment (without extract) as:

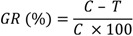

where *C* is the cell concentrations under the control treatment and *T* is the cell concentrations under the extract treatment. Three replicates were considered. The results were recorded as means ±SD of the duplicate experiment. Differences between means of data were compared by least significant difference (LSD) calculated using the Statistical Analysis System (S.A.S., Institute, Inc. Cary, NC, USA).

### 2.5. Antibacterial Activity in Experimentally Contaminated Vacuum-Packed Meat

*Major psoas* muscles were obtained from beef carcasses at 1 h post-slaughter in a local abattoir, and transported to the laboratory under refrigerated conditions. The outer surface of muscle was sterilized by immersion in 95% ethanol and then burning the residual ethanol on the meat surface. After the aseptic removal of the outer surface, the inner core of the sterile meat was aseptically minced by means of a sterile steel meat grinder (autoclaved at 121 °C for 15 min).

Minced meat was also examined for any contamination by bacteria considered in the experiment. Three 10 g subsamples, taken in aseptic conditions after surface sterilization by U.V. light (2 min for each side of the sample) in biohazard hood at 50 cm distance, were placed in stomacher bags and inoculated with a single strain (ca 10^4^ cfu/mL).

The samples were then homogenized in a stomacher (Lab Blender 400, Servard, London, UK) with sterile peptonate water (0.1% peptone and 0.85% NaCl in a 1/10 ratio) for 2 min at room temperature to ensure proper distribution of the bacteria. NCE (10 µg) was added to each inoculated bags. Sterile distilled water and ciprofloxacin were also used as controls. To attain uniform distribution of the added compounds, treated meat samples were further homogenized in a stomacher (Lab Blender 400), as previously described. All stomacher bags with samples from all treatments were sealed under vacuum (Multivac, Type A300/16, Wolfertschenden, Germany) and stored at 10 °C for 12 days, simulating an abusive refrigerated storage.

Detection and identification of bacteria from experimentally treated samples were carried out using molecular biology and microbiological techniques at two day intervals up to the 12th day of refrigerated storage.

#### 2.5.1. Molecular Biology Analysis

Cell detection only based on PCR is a rapid and highly sensitive alternative method, but it is not able to distinguish live from dead cells [20]. The dye propidium monoazide (PMA^TM^ Biotium Inc. Hayward, CA, US) is a photo-reactive dye with high affinity for DNA. The dye intercalates into DNA and forms a covalent linkage upon exposure to intense visible light. It is cell membrane impermeable. When a sample comprising both live and dead bacteria is treated with PMA^TM^, only dead cells are susceptible to DNA modification due to their compromised cell membranes. Thus, it permits selective detection of the live cells, providing a useful count of viable ones among the total amount of bacteria [21,22].

#### 2.5.2. DNA Extraction

The big debris of stomacher samples in suspensions were allowed to deposit for 1 min. Then 12 mL of supernatant was put into screw-cap tubes with PMA^TM^ and placed 25 cm away from a 600 W halogen lamp swirling on ice for 5 min. Then, 0.3 g of glass beads were added in each tube for DNA extraction.

Tubes were subjected to centrifugation at 4 °C for 10 min at 14,000 × g to pellet the cells, whichwere resuspended in 150 µL of proteinase K buffer (50 mM Tris-HCl, 10 mM EDTA (pH 7.5), 0.5% (wt/v) sodium dodecylsulfate). Proteinase K (25 mg/mL; Sigma, Milano, Italy) was added, and a 65 °C treatment was performed for 1.5 h. After this step, 2× breaking buffer (150 µL, 4% (v/v) Triton X-100, 2% (wt/v) sodium dodecylsulfate, 200 mM NaCl, 20 mM Tris (pH 8), 2 mM EDTA (pH 8)) was mixedin the tubes. phenol-chloroform-isoamyl alcohol mixture (300 µL, 25:24:1, pH 6.7; Sigma) for DNA extraction were added to the tubes.

Then, three 30-s treatments at the maximum speed, with an interval of 10 s each, were performed in a bead beater (Fast Prep; Bio 101, Vista, CA, USA). The tubes were then centrifuged at 12,000 × g at 4 °C for 10 min, and the aqueous phase was precipitated with 1 mL of ice-cold absolute ethanol. The DNA was collected at 14,000 × g at 4 °C for 10 min, and the pellets were dried under *vacuum* at room temperature. 50 µL of sterile water was added, and a 30-min period at 45 °C was used to facilitate the nucleic acid solubilisation. 1 µL of DNase-free RNAase (Roche Diagnostics, Milan, Italy) was added to digest RNA by incubation at 37 °C for 1 h.

#### 2.5.3. Species-specific Polymerase Chain Reaction (PCR) and Multiplex PCR Detection

PCR reactions were performed in 50 μL samples containing approximately 50–100 ng of bacterial genomic DNA solution, 5 μL of 10× PCR reaction buffer, 200 μM of each dNTP, 2 mM of MgCl_2_, 0.5 μM of each primer (*Escherichia coli*: Ec1(5′-CCGATACGCTGCCAATCAGT)/Ec2 (5′-ACGCAGA CCGTAAGGGCCAGAT); [23] *Campylobacter jejuni*: hipO-F(5′-GACTTCGTGCAGATATGGATG CTT)/hipO-R(5′-GCTATAACTATCCGAAGAAGCCATCA) and 16S-F(5′-GGAGGCAGCAGTA GGGAATA)/16S-R(5′-TGACGGGCGGTGAGTACAAG) [24]; *Staphylococcus aureus*: 16s 1(5′-CAGCTCGTGTCGTGAGATGT)/16s 2(5′-AATCATTTGTCCCACCTTCG) [25]; Lactic acid bacteria: Y1(TGGCTCAGAACGAACGCTGGCCCG)/Y2 (CCCACTGCTGCCTCCCGTAGGAGT); *Lactobacillus sakei*: 16 (GCTGGATCACCTCCTTTC)/Ls(ATGAAACTATTAAATTGGTAC); *Lactobacillus curvatus*: 16 (GCTGGATCACCTCCTTTC)/Lc(TTGGTACTATTTAATTCTTAG); *Leuconostoc* spp.: Lu1r (CCACAGCGAAAGGTGCTTGCAC)/Lu2(GATCCATCTCTAGGTGACGC CG); *Carnobacterium* spp.: Cb1 (CCGTCAGGGGATGAGCAGTTAC)/Cb2r(ACATTCGGAAACG GATGCTAAT) [26] and 0.5 U of *Taq* Polymerase (Amersham Pharmacia Biotech, Freiburg, Germany) using a model 2400 DNA thermal cycler (Perkin Elmer Corp. Applied Byosystems Division, Monza, Italy).

The primer pairs used for PCR detection and identification are those reported in the literature. Amplified products (7 µL) were analyzed by electrophoresis in 2% or 3% agarose gels buffered in 0.5× TBE (TBE buffer: 90 mM tris(hydroxymethyl)aminomethane, 90 mM boric acid, 3 mM ethylediaminetetraacetate Na salt, pH 8,3) against a 50 bp, 100 bp and 1 Kb ladder used as size marker (Invitrogen, Milano, Italia) and visualized by UV light at 260 nm (Fotodine 3-3102 Celbio, Milano, Italy) after staining with ethidium bromide.

#### 2.5.4. Microbiological Analysis

Microbiological analysis of non-inoculated and inoculated mixed samples was done at 2 day intervals up to the 12th day of refrigerated storage. Each sample was homogenized for 1 min in sterilized peptone water (0.1%) using a stomacher. From this mixture, serial dilutions were prepared and surface plated (100 µL, in duplicate) on agar media and temperatures as follows before presumptive colonies were counted.

For *Campylobacter* spp. detection, the ISO 10272:1995 procedure [27], was followed. Suitable volumes of eluent to *Campylobacter* Enrichment Broth (BHI + *Campylobacter* Growth Supplement and modified Karmali Selective Supplement, OXOID, Rodano, MI, Italy) were added then plated on selective media (Skirrow agar, Karmali agar, Preston Agar, OXOID) and incubated for 48 h at 42 °C in microaerophilic conditions (“Campygen” system, OXOID).

LAB detection was according to ISO/TC 34/SC 6/WG 15, 3 E 5 (1984) [28]. Meat samples were plated on MRS agar pH 5.6 (De Man, Rogosa, Sharpe OXOID) and incubated for 48–72 h at 25 °C and 5–10% CO_2_ in an anaerobic system (BBL Becton Dickinson Co., Buccinasco, MI, Italy).

The control strains were grown on media and at the growth conditions as reported on products sheets. To prepare working cultures, stock cultures were standardized through two successive 24 h growth cycles in the appropriate broth (BHI or MRS) at 20 °C without agitation.

## 3. Results

The growth inhibition zone (IZ) values (mm) obtained by the agar disc diffusion method showed that the NCE had a broad spectrum of antibacterial activity. *Lactobacillus sakei* was the most sensitive (IZ 22.67 mm), followed by *Leuconostoc* spp., C*ampylobacter jejuni* (IZ 21.00 mm), and S*taphylococcus aureus* (IZ 20.00 mm), with *Escherichia coli* being the less susceptible (IZ 11.33 mm). 

There was a significant difference (*p* ≤ 0.05) between the antibacterial activity of NCE (100 µg) (IZ range 11.33–22.67 mm) and the antibiotic activity of ciprofloxacin (IZ range 23.41–32.67 mm) but there was no significant difference between the treatment with NCE and the antibiotic against *C. jejuni* and *Leuconostoc* spp. (*p* ≥ 0.05) (Table 1).

**Table 1:** Antimicrobial activity of NCE against zoonotic and spoilage bacteria detected by disc diffusion method as inhibition zone of growth (mm).

Inhibition zone (mm)				
Bacteria	Treatment			
	NCE	TWN	CFX	WTR
*Escherichia coli*	11.33 ± 0.58b	‒	28.00 ± 1.00a	‒
*Enterococcus faecalis*	14.33 ± 1.15b	‒	25.27 ± 1.00a	‒
*Staphylococcus aureus*	10.00 ± 1.00b	‒	30.33 ± 1.73a	‒
*Campylobacter jejuni*	21.00 ± 1.73a	‒	25.80 ± 1.15a	‒
*Carnobacterium* spp*.*	16.33 ± 0.58b	‒	23.41 ± 1.00a	‒
*Lactobacillus curvatus*	17.50 ± 0.50b	‒	27.33 ± 2.08a	‒
*Lactobacillus sakei*	22.67 ± 0.58c	‒	32.67 ± 2.89a	‒
*Leuconostoc* spp.	21.00 ± 2.00a	‒	25.00 ± 0.00a	‒

NCE = neem cake ethylacetate extract; TWN = Tween^®^ 80; CFX = ciprofloxacin; WTR = sterile distilled water; ‒: Absence of inhibition zone; Values expressed as mean ± Standard Deviation of two experiments. Mean values with different letter in the row are significantly different (*p* ≤ 0.05).

The bacterial growth reduction (%), determined in liquid medium with or without NCE, ranged from 60 ± 1.33–68 ± 0.89 at 0.001/200 µg/µL, 61 ± 1.00–70 ± 0.00 at 0.1/200 µg/µL, 62 ± 1.73–71 ± 1.00 at 1/200 µg/µL to 89 ± 1.00–92 ± 3.21 at 10/200 µg/µL of NCE (Table 2). No significant difference (*p* ≥ 0.05) was detected among the percentages of growth reduction of all tested bacteria when treated with 10 µg of NCE.

**Table 2:** Bacterial growth inhibition (%) at 24 h in liquid medium with different concentrations of NCE using as reference the control treatment (without NCE).

Bacterial growth reduction (%)				
Bacteria	Treatment			
	NCE (10 µg)	NCE (1 µg)	NCE (0,1 µg)	NCE (0.001 µg)
*Escherichia coli*	89.75 ± 1.53**d**	70.61 ± 1.00**bc**	67.67 ± 1.33**b**	60.811 ± 2.08**a**
*Enterococcus faecalis*	90.61 ± 1.15**d**	69.70 ± 1.00**bc**	67.58 ± 1.33**ab**	64.86 ± 1.00**a**
*Staphylococcus aureus*	91.80 ± 3.21**d**	65.81 ± 1.00**bc**	63.74 ± 0.00**ab**	59.60 ± 1.33**a**
*Campylobacter jejuni*	88.88 ± 0.61**d**	61.62 ± 1.73**abc**	60.81 ± 1.00**ab**	61.72 ± 2.03**a**
*Carnobacterium* spp.	88.90 ± 1.00**d**	68.799 ± 1.00**abc**	69.60 ± 0.00**ab**	66.68 ± 1.20**a**
*Lactobacillus curvatus*	90.73 ± 1.53**d**	69.70 ± 2.08**bc**	68.69 ± 2.00**b**	62.83 ± 1.73**a**
*Lactobacillus sakei*	89.81 ± 1.00**d**	69.61 ± 0.58**abc**	69.57 ± 0.00**ab**	67.58 ± 0.89**a**
*Leuconostoc* spp.	89.71 ± 0.29**d**	65.66 ± 1.00**bc**	67.81 ± 0.58**ab**	60.71 ± 0.58**a**

NCE = neem cake ethyl acetate extract; Values expressed as mean ± Standard Deviation of two experiments (three repetitions for each experiment). Mean values with different letters in the column are significantly different (*p* ≤ 0.05).

Amplicons of expected sizes of species-specific genomic sequences of all tested bacteria were obtained for all bacteria tested and those used as controls. They were always detected in the control samples (water and Tween 80^®^) at 2nd, 4th, 6th, 8th, 10th, and 12th storage days. They were never detected in samples treated with ciprofloxacin collected on the same storage days. While they were revealed in all the samples treated with NCE at 2nd and 4th storage day. *E. coli* and *C. jejuni* were also detected at the 6th and 8th storage day. *S. aureus* was detected on all storage days (Table 3).

**Table 3:** Detection and identification at 2, 4, 6, 8, 10 and 12 days after treatment with NCE (10/200 µg/µL) of the tested bacterial strain viable cells in vacuum packed minced beef meat stored at 10 °C.

Storage day																		
Bacteria	Treatment																	
	2			4			6			8			10			12		
	NCE	W	CFX	NCE	W	CFX	NCE	W	CFX	NCE	W	CFX	NCE	W	CFX	NCE	W	CFX
*Escherichia coli*	+	+	−	+	+	−	+	+	−	+	+	−	−	+	−	−	+	−
*Enterococcus faecalis*	+	+	−	+	+	−	−	+	−	−	+	−	−	+	−	−	+	−
*Staphylococcus aureus*	+	+	−	+	+	−	+	+	−	+	+	−	+	+	−	+	+	−
*Campylobacter jejuni*	+	+	−	+	+	−	+	+	−	+	+	−	−	+	−	−	+	−
*Carnobacterium* spp.	+	+	−	+	+	−	−	+	−	−	+	−	−	+	−	−	+	−
*Lactobacillus curvatus*	+	+	−	+	+	−	−	+	−	−	+	−	−	+	−	−	+	−
*Lactobacillus sakei*	+	+	−	+	+	−	−	+	−	−	+	−	−	+	−	−	+	−
*Leuconostoc* spp.	+	+	−	+	+	−	−	+	−	−	+	−	−	+	−	−	+	−

+ = detected; − = not detected; NCE = neem cake ethylacetate extract; W = sterile distilled water; CFX = Ciprofloxacin.

The microbiological detections of microorganisms by official methodologies were in agreement with the molecular biology methodology detections carried out as previously described at each interval to test the antibacterial activity in experimentally contaminated vacuum-packed meat. Numbers of bacterial cells were converted to log cfu and subjected to statistical analyses. Three replicates were considered. The results were recorded as means ±SD of the duplicate experiment. Differences between means of data were compared by least significant difference (LSD) calculated using the Statistical Analysis System (S.A.S., Institute, Inc. Cary, NC, USA). The numbers of viable bacterial cells never significantly (*p* ≤ 0.05) overcome the inoculums’ concentration used to experimentally contaminate meat at each interval considered (Table 4).

**Table 4:** Bacteria counts obtained using the official microbiological analysis at 2 days intervals up to the 12th day of refrigerated storage to test the antibacterial activity in experimentally contaminated vacuum-packed meat.

Bacterial counts (log_10_ CFU/mL)							
Bacteria	Storage days						
	0	2	4	6	8	10	12
*Escherichia coli*	6.87 ± 0.5a	6.90 ± 0.5a	6.93 ± 0.5a	6.91 ± 1.2a	6.90 ± 1.5a	0.00	0.00
*Enterococcus faecalis*	7,23 ± 1.0a	7.30 ± 0.0a	7.28 ± 1.0a	0.00	0.00	0.00	0.00
*Staphylococcus aureus*	7.54 ± 2.5a	7.60 ± 0.5a	7.63 ± 0.5a	7.65 ± 1.5a	7.60 ± 2.0a	7.61 ± 0.5a	7.63 ± 1.5a
*Campylobacter jejuni*	7.20 ± 1.0a	7.28 ± 1.3a	7.26 ± 0.5a	7,23 ± 1.8a	7.21 ± 1.0a	0.00	0.00
*Carnobacterium* spp.	7.69 ± 2.0a	7.75 ± 0.5a	7.71 ± 0.0a	0.00	0.00	0.00	0.00
*Lactobacillus curvatus*	6.95 ± 1.0a	7.30 ± 0.5a	7.21 ± 1.9a	0.00	0.00	0.00	0.00
*Lactobacillus sakei*	7.20 ± 0.0a	7.49 ± 0.0a	7.28 ± 1.0a	0.00	0.00	0.00	0.00
*Leuconostoc* spp.	6.97 ± 1.5a	7.11 ± 0.0a	7.20 ± 0.5a	0.00	0.00	0.00	0.00

Values expressed as mean ± Standard Deviation of three repetitions. Mean values with different letter in the row are significantly different (*p* ≤ 0.05).

This evidence should suggest that NCE acts mainly as a bacteriostatic agent. It successfully displays bactericidal effects against all bacteria tested, with the exception of *Staphylococcus aureus*. A critical step in the development of novel classes of antibacterial agents is the limited understanding of the molecular mechanism of action (MOA) of bioactive substances. Genome-wide expression technologies coupled with advanced data analysis techniques, may help to elucidate the bacterial defense mechanisms against antibiotic stress and in the same time to elucidate the mechanism of action of antibacterial activity of natural substances.

**Figure 2:**
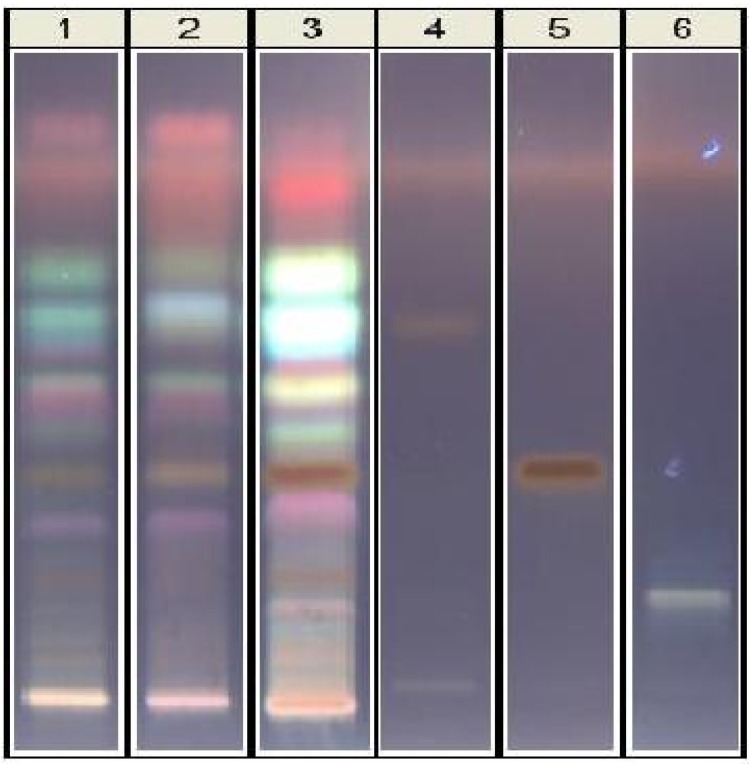
HPTLC analysis of neem cake extracts and selected standards of nortriterpenes. Tracks: **1**, methanol extract after 24 h; **2**, methanol extract after 72 h; **3**, ethylacetate extract; **4**, nimbin; **5**, salannin; **6**, azadirachtin A.

The chemical analysis showed, beside the presence of azadirachtins, other constituents were also present, including a predominant mixture of fatty acids methyl esters. The determination of active constituents of NCE must be achieved. Thus, HPTLC analyses showed a great complexity of NCE composition [29], although neem nortriterpenes were first considered as the active principles, other constituents must be tested, as well as other types of extracts. The HPTLC technique is able to show the differences in composition on NCE in comparison with the methanol extract of neem cake. In the HPTLC plate (Figure 2), in comparison with selected standards, tetranortriterpenes are present but not predominant, as confirmed by HPLC, with salannin as the main limonoid, whereas azadirachtin A is the main one in the oil. On the contrary, highly fluorescent compounds are very evident on the upper side of the plate. The identities of these substances must be determined. The quantitative results in main limonoids were (in ppm): azadirachtin A 79, nimbin 26, salannin 858, as already reported [30].

## 4. Discussion and Conclusions

Nowadays meat quality is considered the result of a co-occurrence of evident and cryptic characters, heavily based on conservation efficacy. The microbial safety and stability, as well as the sensory and nutritional quality of most foods, are based on application of combined preservatives factors.

The antibacterial activity of neem products is known, being mainly focused on the antimicrobial properties of azadirachtin A. However, whereas in neem oil azadirachtins account for the main constituents, in neem cake salannin is predominant. Attention on neem cake was so far focused on soil microflora effects, due to its predominant use as a fertilizer [31,32,33]. This study, owing to its low cost and the antimicrobial potentiality of neem, considered a selected neem cake extract as a model for exploring antibacterial, the use of natural substances as well as their derived commercial products, with a view to mass treatment of meat products. NCE has a characteristic odor that at the amount used in the experiment was not perceived. Anyway it is necessary to make organoleptic tests to demonstrate it has no sensory effect on treated meat.

In this regard, owing to the particular characters of plant natural products, an effort to produce new methods for testing their antibacterial activity is crucial, tailoring the most recent analytical approaches, rooted in molecular biology and seeking urgent help from metabolomic methods and systems biology, beside official methods as reported in literature [34,35,36]. Bacterial adaptation ability will only be stopped successfully by exploring new pathways.

In this research data were used to support the evidences of the target activity, consisting in developing an alternative antibacterial product to be used in fresh or in cooked cured retail meat products. To be suitable the antibacterial product should be low cost, eco-friendly, target tailored, besides being effective. The obtained results demonstrate that NCE counteracts the growth of Gram-negative, Gram-positive and microaerophilic bacteria. Optimal concentrations for its use in food preservation technologies should be found to ensure the safety of the food, with appropriate organoleptic characteristics, and acceptable to consumers. Studies aiming to elucidate the interaction between NCE and components of fresh retail meat matrices or additives as well the stability of NCE during meat processing and packaging are still needed.

## References

[1] Hassan M.M., Oyewale A.O., Amupitan J.O., Abduallahi M.S., Okonkwo E.M. (2004). Preliminary Phytochemical and antibacterial investigation of crude extracts of the root bark of *Detarium microcarpum*. J. Chem. Soc. Nigeria.

[2] Usman H., Abdulrahman F.I., Usman A. (2009). Qualitative phytochemical screening and *in vitro* antimicrobial effects of methanol stem bark extract of *Ficus thonningii* (*Moraceae*). Afr. J. Tradit. Complement. Altern. Med..

[3] Solomakos N., Govaris A., Koidis P., Botsoglou N. (2008). The antimicrobial effect of thyme essential oil, nisin, and their combination against *Listeria monocytogenes* in minced beef during refrigerated storage. Food Microbiol..

[4] Palanappian K., Holley R.A. (2010). Use of natural antimicrobials to increase antibiotic susceptibility of drug resistant bacteria. Int. J. Food Microbiol..

[5] Ghosh S., Lapara T.M. (2007). The effect of subtherapeutic antibiotic use in farm animals on the proliferation and persistence of antibiotic resistance among soil bacteria. Int. Soc. Microb. Ecol. J..

[6] Hammerum A.M., Heuer O.E. (2009). Human health hazards from antimicrobial resistant *Escherichia coli* of animal origin. Clin. Inf. Dis..

[7] World Health Organization/United Nations Environment Programme (WHO/UNEP) (1989). Public Health Impact of Pesticides Used in Agriculture.

[8] Abbasi P.A., Riga E., Conn K.L., Lazarovits G. (2005). Effect of neem cake soil amendment on reduction of damping-off severity and population densities of plant-parasitic nematodes and soilborne plant pathogens. Can. J. Plant Pathol..

[9] Ogbuevo I.P., Odoemenam V.U., Obikaonu H.O, Opara M.N., Emenalom O.O., Uchegbu M.C., Okoli I.C., Esonu B.O., Floeje M.U. (2011). The growing importance of neem (*Azadirachta indica* A. Juss) in agriculture, industry, medicine and environment: A Review. Res. J. Medic. Plant.

[10] Rao V.K., Kowale B.N., Verna A.K. (2003). Effect of feeling washed neem (*Azadirarachta indica*) seed kernel cake on the quality, lipid profile and fatty acid composition of goat meat. Small Rumin. Res..

[11] Baswa M., Rath C.C., Dash S.K., Mishra R.K. (2001). Antibacterial activity of karanj (*Pongamia pinnata*) and neem (*Azadirachta indica*) seed oil: A preliminary report. Microbios.

[12] Ram M.S., Ilavazhagan G., Sharma S.K., Dhanraj S.A., Suresh B. (2002). Anti-microbial activity of a new vaginal contraceptive NIM-76 from neem oil (*Azadirachta indica*). J. Ethnopharmacol..

[13] Grare M., Fontanay S., Cornil C., Finance C., Duval R.E. (2008). Tetrazolium salts for MIC determination in microplates: Why? Which salt to select? How?. J. Microbiol. Methods.

[14] Rahman M., Kuhn M., Rahman P., Olsson-Liljequist B., Molby R. (2004). Evaluation of a scanner-assisted colorimetric MIC method for susceptibility testing of Gram-negative fermentative bacteria. Appl. Environ. Microbiol..

[15] Klančnik A., Piskernik S., Jeršek B., Smole Možina S. (2010). Evaluation of diffusion and dilution methods to determine the antibacterial activity of plant extracts. J. Microbiol. Methods.

[16] Make the Planet Safer and Greener with GreeNeem. Virudhunagar, India. www.greeneem.com.

[17] Nicoletti M., Serafini M., Aliboni A., D’Andrea A., Mariani S. (2010). Toxic effects of neem cake extracts on *Aedes albopictus* (Skuse) larvae. Parasitol. Res..

[18] Del Serrone P., Contillo R., Failla S., Della C.G., Lanni L., Saccani G., Saccares S. (2006). Assessment of the microbiological quality of retail fresh pork meat in central Italy. Ital. J. Food Sci..

[19] Del Serrone P., Saccares S., Saccani G. Identification of Microorganisms Affecting Quality of Retail Fresh Pork Meat using Microbiological and Molecular Techniques. Proceedings of the Atti 3rd CIGR—Food and Agricultural Products Processing and Innovations.

[20] Frantanico P.M., Bhunia A.K., Smith J.L. (2008). Foodborne Pathogens: Microbiology and Molecular Biology.

[21] Nocker A., Cheung C.Y., Camper A.K. (2006). Comparison of propidiummonoazide with ethidiummonoazide for differentiation of live *vs.* dead bacteria by selective removal of DNA from dead cells. J. Microbiol. Meth..

[22] Chen S., Wang J., Stein B.C., Ge R.E. (2011). Rapid detection of viable Salmonellae in produce by coupling propidiummonoazide with loop-mediated isothermal amplification. Appl. Environ. Microbiol..

[23] Osek J. (2001). Multiplex polymerase chain reaction assay for identification of enterotoxigenic *Escherichia coli* strains. J. Vet. Diagn. Invest..

[24] Linton D., Lawson A.J., Owen R.J., Stanley J. (1997). PCR detection, identification to species level, and fingerprinting of *Campylobacter jejuni* and *Campylobacter coli* direct from diarrheic samples. J. Clin. Microbiol..

[25] Martineau P., Roy P.M., Bergeron M.G. (1998). Species-specific and ubiquitous-DNA-based assay for rapid identification of S*taphylococcus aureus*. Clin. Microbiol..

[26] Yost C.K., Nattress F.M. (2000). The use of multiplex PCR reactions to characterize populations of lactic acid bacteria associated with meat spoilage. Lett. Appl. Microbiol..

[27] (1995). Microbiology of Food and Animal Feeling Stuffs. Horizontal Methods for Detection of Thermotolerant Campylobacter. International Standard ISO 10272:1995 (E).

[28] (1984). Enumeration of *Lactobacteriaceae* in Meat and Meat Products.

[29] Nicoletti M., Mariani S., Maccioni O., Coccioletti T., Murugan K. (2012). Neem cake: Chemical composition and larvicidal activity on Asian tiger mosquito. Parassitol. Res..

[30] Nicoletti M., Maccioni O., Coccioletti T, Mariani S., Vitali F., Perveen F. (2011). Neem Tree (*Azadirachta indica* A. Juss) as Source of Bioinsecticides. Insecticides—Advances in Integrated Pest Management.

[31] Atawodi S.E., Atawodi J.C. (2009). *Azadirachta indica* (neem): A plant of multiple biological and pharmacological activities. Phytochem. Rev..

[32] Stalk J.D., Walter J.F. (1995). Persistence of azadirachtin A and B in soil: Effect of temperature and microbial activity. J. Environ. Sci. Health.

[33] Gajalakshmi S., Abbasi S.A. (2004). Neem leaved as a source of fertilizer-cum pesticide vermicompost. Bioresour. Technol..

[34] Hecht D.W., Aldridge K.E., Citron D.M., Cox M., Webb C.D., Jacobus N., Wexler H.M., Jenkins S.G., Onderdonk A., Aldridge K.E., Roe-Carpenter D., Citron D.M., Rosenblatt J.E. (2000). National Committee for Clinical Laboratory Standards Methods for Dilution Antimicrobial Susceptibility Tests for Bacteria That Grow Aerobically.

[35] (2009). European Committee on Antimicrobial Susceptibility Testing EUCAST. Disk Diffusion Method for Antimicrobial Susceptibility Testing. www.eucast.org.

[36] Lalitha M.K. (2011). Manual on Antimicrobial Susceptibility Testing. http://www.ijmm.org/documents/Antimicrobial.doc.

